# Perceptions of Brazil’s Bolsa Família cash transfer programme, life opportunities and mental health in the lives of young adults from the outskirts of São Paulo: qualitative study

**DOI:** 10.1192/bjo.2025.10056

**Published:** 2025-06-30

**Authors:** Paulo Malvasi, Sara Evans-Lacko, Eva Cyhlarova, Alicia Matijasevich, David McDaid, Cristiane Silvestre Paula

**Affiliations:** Department of Public Health, Faculty of Medical Sciences of Santa Casa de São Paulo, São Paulo, Brazil; Care Policy and Evaluation Centre, London School of Economics and Political Science, London, UK; Department of Preventive Medicine, Faculty of Medicine FMUSP, University of São Paulo, São Paulo, Brazil; Human Developmental Sciences Graduate Program, and Mackenzie Center for Research in Childhood and Adolescence, Mackenzie Presbyterian University, São Paulo, Brazil

**Keywords:** Youth psychiatry, qualitative research, monetary incentive, mental health, vulnerable populations

## Abstract

**Backgound:**

Cash transfer programmes (CTPs) provide financial support to alleviate poverty and promote economic stability. The Bolsa Família Programme (BFP), a Brazilian initiative and the world’s largest CTP by number of beneficiaries, aims to improve living conditions. While poverty is closely linked to poor mental health, evidence regarding the specific effects of CTPs on young adults’ mental health remains limited, underscoring the need for further research.

**Aims:**

To understand the meaning attributed to the BFP by young adults regarding their future aspirations and mental health, as well as perspectives from providers.

**Method:**

This qualitative study was conducted at the outskirts of São Paulo city, involving 12 in-depth interviews with young adults aged 18–24 years and 2 focus groups comprising 17 health and social assistance professionals.

**Results:**

Thematic analysis identified four themes according with interviews and focus groups: (a) perceptions about poverty (hopelessness and lack of opportunities); (b) impact of poverty on mental health (anxiety, unpredictability and hopelessness as consequences of living in poverty); (c) young adults’ needs and aspirations (job opportunities as the main expectation for a better future); and (d) BFP limitations and opportunities for improvement (BFP perceived as just one of the survival strategies but not impacting life opportunities for young adults).

**Conclusions:**

The BFP was valued as essential for meeting poor families’ basic needs. Employment opportunities were central to young adults’ expectations, often causing anguish and anxiety. Expanding the BFP to include employment and income-generation policies could better support the mental health and life opportunities of vulnerable youth.

Poverty is a multifaceted issue involving interconnected factors such as economic hardship, social exclusion and limited access to education and healthcare. Addressing this requires a comprehensive understanding of its effects on both individuals and communities.^1,2^ Numerous studies have shown that poverty and social inequality are associated with poor mental health.^3^ Stress and environmental conditions associated with poverty, as well as the low probability of receiving specialised treatment,^4,5,6^ heighten the risk of mental health problems. At the same time, mental health problems can increase the risk of future poverty due to the higher expenses related to healthcare, diminished productivity and problems with economic decision-making, stigmatisation surrounding mental illness and the loss of educational and employment opportunities with a subsequent decline in earnings.^7,8^

Evidence from low -and middle-income countries (LMICs) suggests that poverty is associated with greater risk of developing mental health problems in young people,^9,10^ because they face a range of concurrent deprivations^2^ and vulnerable situations such as exposure to different types of violence^11^ and a lack of educational and employment opportunities, which significantly limit their life chances.^12–14^ At the same time, living in poverty leads to worse mental health, reducing life chances and perpetuating poverty – establishing a vicious circle that can be particularly negative during youth and early adulthood.^15^

## Cash transfer programmes

Cash transfer programmes (CTPs) are anti-poverty initiatives that provide direct financial support to vulnerable individuals or families. Their primary goal is to alleviate poverty, reduce social inequality and empower beneficiaries to meet basic needs such as food, education and healthcare,^8^ ultimately improving people’s quality of life.

Over the past three decades, CTPs^16^ have been a key strategy for reducing poverty and income inequalities in LMICs, showing consistent improvements in health,^17^ poverty and educational, suicide and homicide outcomes.^18^ Although there is growing interest in understanding their impact on mental health, most studies have focused on adults, with varied or inconclusive findings.^19,20^ While CTPs have been shown to improve aspects of adult mental health in some LMICs (e.g. reducing suicide rates in Brazil^21^), evidence in regard to young people remains mixed, with some studies reporting reductions in depressive symptoms while others show no impact.^20,22,23,24^

A recent systematic review showed that CTPs alone may not be enough to improve the mental health of children and young people living in poverty,^20^ even though CTPs combined with psychosocial interventions can have positive effects on the lives of children and young people in the long term.^25,26^ There is limited knowledge about the mechanisms by which CTPs can affect the mental health of young people, but factors influencing the outcomes may include the types of cash transfer policies, the amount of money transferred and the age of the target population.^8,27^ In the short term, CTPs have shown evidence of improving food security and reducing stress related to financial instability. In the long term, they can foster economic security, strengthen social relationships, improve access to education and encourage future investments.^28^

## Bolsa Família programme

In 2003, the Brazilian federal government established the Bolsa Família programme (BFP). This provides a minimum level of income to poor families in Brazil with at least one child aged 0–17 years, as well as to pregnant women.^18,29^ Families living in extreme poverty (monthly income up to ∼USD16 per person) receive unconditional cash transfers, while those living in poverty (monthly income ∼USD16–33 per person) receive conditional transfers. These conditionalities include health check attendance, child vaccinations and school participation.^18^ A case-control study of 1266 children (66% BFP beneficiaries) found that those aged 7–17 years and receiving BFP reported better psychosocial health, including satisfaction with friendships and behaviour, compared with their peers.^30^ Conversely, a cohort study of 2063 BFP beneficiaries found no association between BFP participation in childhood and mental health outcomes in early adolescence.^31^

In this paper, we present the results of a qualitative study that was part of the international multisite project ‘Poverty reduction, mental health and the chances of young adults: mechanisms through analysis from 6 low- and middle-income countries’ (CHANCES-6). The project’s main objective was to advance current understanding of the dynamics among poverty, mental health and life chances, particularly of young adults, examining the impact of poverty reduction policies on mental health. The project includes qualitative and quantitative studies across six LMICs: Brazil, Colombia and Mexico in Latin America, and South Africa, Liberia and Malawi in Africa. In Brazil, the CHANCES-6 project focuses on evaluating the impact of the BFP on the mental health of children and adolescents. The current study presents the full qualitative findings from the Brazilian arm of the CHANCES-6 project.^32^

## Objectives

The aims of this study were to understand the views and experiences of young adults and the perspectives of service providers regarding the delivery of CTPs. Specifically, we explored (a) young adults’ experiences and perceptions of poverty and mental health and their involvement in the BFP; (b) their aspirations for the future; (c) the perceived role of the BFP and strategies to support youth while addressing poverty and mental health, based on the perspectives of both young adults and service providers; and (d) barriers to current programme delivery and opportunities for improvement, as identified by young adults and service providers.

## Method

### Study design

In this qualitative study, we conducted face-to-face focus groups and individual in-depth interviews. We combined two research strategies to capture the broader social and experiential aspects of the study, ensuring diversity of perspectives. Service providers contextualised the life circumstances of BFP beneficiaries based on their professional experience, while young adults shared their experiences of receiving the benefit during childhood and adolescence and its impact on their lives.

### Study settings

Participants were recruited from residents of two neighbourhoods on the outskirts of São Paulo city: Sapopemba and Vila Maria. These regions were chosen due to their differing Human Development Index (HDI): Sapopemba has a low HDI (0.74), while Vila Maria has a medium HDI (0.85), both marked by significant social inequality. Another criterion was spatial location: Sapopemba is situated on the far eastern outskirts of São Paulo, while Vila Maria is in the north zone. Sapopemba spans 13.50 km^2^, with a population of 284 524 and a demographic density of 21 076 inhabitants/km^2^. In comparison, the sub-prefecture of Vila Maria covers 26.40 km^2^, with a population of 297 713 and a demographic density of 16 873 inhabitants/km^2^.

### Sample selection and data collection

The selection of interview participants aimed to represent the characteristics of income, housing and access to public services in these two neighbourhoods on the outskirts of São Paulo: (a) young adults aged between 18 and 24 years and whose families were registered as users of the BFP; and (b) male or female, aiming for 50% of each.

The main criterion for focus group participation was involvement in community activities aimed at combating poverty and expanding opportunities for young adults, including work with non-governmental organisations, public assistance and health services and community projects in the study regions. Participants included professionals such as social workers, community health workers, human rights activists and community leaders. The focus groups brought together individuals with shared professional identities, defined by their collaboration within the same institutions.^33^

Recruitment for both focus groups and interviews was facilitated by providers in the two neighbourhoods, and no exclusion criteria were applied. The professionals also contributed by suggesting additional questions that our team had not previously considered, which were subsequently incorporated into the topic guide.

Potential participants were initially informed about the objectives of the study by professionals who were working in the community. Subsequently, the field researcher contacted them by telephone to discuss participation. On the day of the research, the consent form was read jointly between the researcher and participants. All participants signed a written informed consent. The interviews and focus group were recorded and transcribed.

The authors assert that all procedures contributing to this work comply with the ethical standards of the relevant national and institutional committees on human experimentation, and with the Helsinki Declaration of 1975 as revised in 2013. All procedures involving human subjects/patients were respected, and ethical approval was granted by the National Research Ethics Commission (CONEP; CAEE Process no. 4 06777318.2.0000.0084)

Interviews with young adults explored their perspectives on life, their involvement with the BFP and the meanings they attribute to their context, poverty and related factors. The topic guide was collaboratively developed and adapted by CHANCES-6 researchers from Colombia, South Africa and Brazil. The topic guide covered: (a) general life trajectories; (b) experiences and challenges related to the BFP, including its conditionalities; and (c) their views on expectations for the future, life opportunities and mental health.

The focus groups with providers aimed to explore contextual issues based on their perceptions of young adults’ realities. The topic guide covered: (a) young adults’ daily challenges and poverty, and its effects on their future prospects and mental health; (b) providers’ views on the contributions of the BFP in expanding opportunities for young adults, such as access to better education, professional training and employment; and (c) the potential benefits of combining the BFP with psychological counselling or other services to enhance young people’s mental well-being.

### Participants

In total, 29 individuals participated in the study. Two in-person focus groups were conducted in March 2020 with 17 providers: 7 from Sapopemba (aged 25–68 years) and 19 from Vila Maria. Between October and December 2020, we conducted individual face-to-face interviews with 12 young adults aged 18–24 years and whose families were current or former beneficiaries of the BFP. The sample included seven women and five men, with six participants from each neighbourhood. Each focus group consisted of between six and ten participants, a size that ensured effective facilitation and productive discussion.^34^

Individual interviews were conducted based on the principle of theoretical saturation.^34^ Saturation was reached during the analysis and systematisation of themes at the 11th interview. A 12th interview was conducted to ensure balance between locations and genders, in line with the study design.

### Data analysis

We used thematic analysis to identify patterns of meaning that frequently appeared across our data.^35^ To ensure a coordinated analysis of the different data sources, we triangulated: (a) content from the focus groups with providers, which offered contextual insights into the lives of young adults; and (b) interviews capturing young adults’ perceptions. This analysis identified recurring and unique information within each group, highlighting similarities and differences across the systematised themes.^34^

We adopted a deductive approach to thematic analysis,^36^ starting with predefined codes to organise the text for subsequent interpretation and in-depth data analysis. The principal researcher (P.M.) and research supervisor (C.S.P.) conducted a preliminary reading of the focus group and interview transcripts, and developed the interpretation based on the research question and theoretical framework. The analysis was initially conducted in Portuguese and later translated into English.

The first stage of the thematic analysis, familiarisation with the data, was based on the reading and systematic analysis of the data according to four major predefined themes, which guided the interviews and focus groups: (a) poverty; (b) cash transfer policy (specifically the BFP); (c) life opportunities; and (d) mental health.

In the second stage, a series of initial categories emerged within each of the predefined themes: (a) poverty: misery, social inequality, lack of opportunities and violence; (b) BFP: lack of focus on young adults, mothers’ empowerment, fighting hunger, bureaucracy and low cash value; (c) life opportunities: social injustice, low self-esteem, hopelessness, dreams, unemployment, informal work and crime; (d) mental health: anxiety, anger, aggression, life conflicts, depression, sadness and resilience.

In the third stage, we started from the initial categories that emerged in step two and aimed to identify themes in which these codes could be grouped. We developed detailed notes on the contents and observed recurrent concepts and themes in the data, which were grouped into further categories.

In step four, we reviewed the themes from step three and examined whether they were recurring systematically, with the aim of improving and developing the preliminary themes. Four main themes were identified: (a) perceptions of poverty; (b) the central role of poverty in life expectations and mental health; (c) employment and income opportunities; and (d) BFP limitations as an isolated public policy.

In step five, we refined the themes and aimed to identify the essence of each.^37^ We analysed the data according to the emphasis given by participants. We identified expressive and meaningful sentences that captured the most recurrent perceptions from the interviews and focus groups. We identified the following themes and descriptions: (a) perception of poverty (‘Poverty is when we die inside’); (b) impact of poverty on mental health of young adults (‘There are days when we don’t believe in anything any more’); (c) young adults’ needs (‘A job opportunity is what young adults are looking for!’); and (d) the limitations and potential of BFP (‘What I perceive in this neighbourhood is the politics of misery’).

Data analysis was performed by the field researcher (P.M.), with the supervision of the coordinator of qualitative research in Brazil (C.S.P.) and review by the main researcher of the CHANCES project (S.E.-L.). Data were also presented and discussed at monthly CHANCES Project qualitative team meetings during 2021. This qualitative data analysis was conducted manually, without the use of software.

## Results

The combined results from interviews and focus groups are presented according to the four final themes. Each theme is introduced with contextualisation from the focus groups, followed by the young adults’ perceptions and summaries of the different perspectives of the research participants.

### Theme 1: perception of poverty/different kinds of poverty

#### ‘Poverty is when we die inside’

Focus group participants contextualised the challenges faced by young adults in developing a broader, positive perspective on life, given the realities of their daily lives. They observed that poor young adults often lack dreams or long-term life projects, because their focus is on addressing immediate, basic needs and simply staying alive. According to participants, young adults have few role models to inspire aspirations for life opportunities, because few individuals in their surroundings have achieved significant success in work, income, education or economic stability:

‘I think poverty affects young adults a lot, because it creates frustration, I think maybe he can’t have dreams, life projects. I think it comes from this sequence of frustrations he’s been facing, and then he ends up being immediate, thinking about today, tomorrow at the most’ (S, female, provider, 42 years old, Sapopemba).

In this sense, young adults perceive that living in poverty makes autonomy and the pursuit of life goals beyond survival difficult to achieve:

‘I think a hungry person can’t get anywhere. The worst thing is to see a human being without a plate of food, especially now in this pandemic [of COVID -19], the work has decreased, if there is no help or service, how is Brazil going to make it?’ (A, female, beneficiary, 24 years old, Vila Maria).

Although the meanings of poverty varied, there were two recurrent categories: ‘misery’ and ‘lack of opportunities in life’, including difficulties at school and lack of work. Each of these categories allowed participants to open up and discuss contextual aspects of their lives, families and communities. In the words of the young adults, poverty produces feelings of hopelessness, something that ‘kills from within’:

‘I think poverty for the people is not having anything to eat. I don’t know how to explain it. Like I’m poor, but I’m fine and I have my family. I eat every day. For me poverty is when you die inside, not just when you starve yourself. It ends up affecting himself inside, dying inside, with depression, stuff like that’ (L, female, beneficiary, 19 years old, Sapopemba).

Almost all interview and focus group participants considered misery as the central component of poverty. They defined misery as hunger, homelessness and experiences that harm human dignity. Participants described an important difference between misery and poverty. Poverty was interpreted as a condition of those who are ‘humble’ – a term commonly used to refer to the condition of poverty in the outskirts of São Paulo; and misery was interpreted as the condition of those who do not have food, clothes or minimum means of subsistence:

‘One thing I notice is that the difference is in the city blocks, so you are in a block here, it’s a type of person, with a different quality of life, but if you go to the other side, crossing the street, it’s already changed. You know people [are] afraid of rain, on the other side we’re asking for the rain, and others are afraid of it because it’s going to flood the house, the rats will come, the sewage will rise’ (G, male, provider, 23 years old, Sapopemba).

### Theme 2: impact of poverty on mental health

#### ‘There are days when we don’t believe in anything any more’

According to providers, the social context of poverty intersects with mental health issues in families’ daily lives. Traumatic situations, such as domestic violence, impact mental health but are often not properly addressed due to the pressing challenges associated with poverty:

‘What is the daily life of most of these young adults like? Here we have many situations of young adults with depression, in which the family often does not have time to identify this disease, and most of the time people do not understand it as a disease. And this comes from his childhood, because he grows up in a violent family environment’ (R, female, provider, 55 years old, Sapopemba).

Only 2 of the 12 young adults mentioned a diagnosis of a psychiatric disorder. However, the lack of hope for the future was very common in the statements of all interviewees. Young adults eventually lose hope after a prolonged life trajectory in poverty. Their statements included a lack of faith in government actions and comments about the unequal structure of society but, most notably, they expressed the absence of positive role models in their community. Examples of academic success, professional achievements or economic stability in their families or neighbourhoods were rare. As a result, hopeful aspirations seemed very distant, leading to suffering and frustration, because they recognised that the possibility of achieving better life opportunities was very low:

‘Sometimes we end up giving up because we live in a shanty town and we see how the world is more interested in money than helping, we end up giving up on chasing our dream and getting used to the life we have. Because our address excludes us. We look to the side and who had a chance to grow? I try to close my eyes to the world and get into my head to say “that if I want to, I can”’ (L, female, beneficiary, 19 years old, Sapopemba).

The mental health condition most mentioned, both in focus groups and interviews, was anxiety. This symptom was named and frequently exemplified in concrete situations, such as experiences in schools, job interviews or conversations with some authorities (e.g. school directors, judges, police officers). It was a feeling also commonly mentioned when they spoke about their future, what they would like to do and the difficulties they faced in reaching their goals. They reported feeling anxious about getting their first job and their lack of experience and educational training:

‘Feeling this sadness of not being listened to, of not having opportunities, gets in the way that it ends up killing us inside. There are days when we don’t believe in anything any more, we don’t want to have life or anything anymore. We are afraid to search because we think we don’t know how to talk … If I say something wrong in a job interview, suddenly I feel that they will throw stones on me’ (P, male, beneficiary, 18 years old, Vila Maria).

### Theme 3: young adults’ needs and aspirations

#### ‘A job opportunity is what young adults are looking for!’

In the focus groups, participants highlighted how the lack of employment opportunities for poor young adults was associated with a feeling described as ‘indignation’. Additionally, the discussions emphasised that involvement in crime is often contextualised within the cycle of poverty, which participants referred to as a ‘lack of opportunities’.

In several social organisations in São Paulo city, it is common for beneficiaries who excel in community work to become providers for the institution. Therefore, some of the focus group participants were concurrently beneficiaries of the BFP. The statement below is representative of this hybrid position and highlights a clear understanding of the importance of employment and income opportunities for young adults and their families:

‘My son is 14 years old; he already has a profession; my son has been a barber since he was 14 years old. So, he had an opportunity, but he did not break this opportunity into a possibility of growth. Bolsa Família improved the issue of food, clothing, because I was never able to give away a R$1500 shoe, he bought these shoes when he went to work in drug trafficking. So, from the moment I have an opportunity, but through that opportunity it doesn’t become possible, that’s automatically where the crime wins’ (F, male, provider, 45 years old, Vila Maria).

One of the most recurrent narratives was what happened when young adults searched for a job and employers asked about their previous experience, which they did not have. They felt that they lived in a vicious circle of unemployment from which it was difficult to get out:

‘That’s what young adults are looking for, a job opportunity. Nowadays you go after a job, but if you don’t have previous experience, you don’t get it. If Brazil does not provide opportunities for us young adults, how are we going to have any experience someday for anything?’ (A, female, beneficiary, 24 years old, Vila Maria).

Of the 12 young adults interviewed, only 1 was attending university and working as a community health worker. His account illustrates the issues related to dreams and life chances that young adults on the outskirts of São Paulo would like to have:

‘You asked about dreams; I think about my own trajectory … The only thing I had at home was to go to public school. My career plans were at best to be a supermarket manager, which I saw in the people around me, it was that kind of work. My mother works as a supermarket cashier, my uncle is a construction worker, and then I never had anyone to help me to see beyond, that was when I came here to the Centre for the Defence of Children and Adolescents [CEDECA]. I started taking a dance class, I learned to walk around São Paulo, which I didn’t know. I didn’t know how to take the bus alone, I started to meet people who brought me dreams beyond, today I’m studying Psychology. I’m in the second year of Psychology, it is because I found a space where it brings all this knowledge to me, and open doors to other places, to other people. I think this issue, education, health, lack of places for culture, leisure, information, I think this is important, it ends up bringing dreams to young adults’ (G, male, provider, 23 years old, Sapopemba).

Participants in both the focus groups and interviews emphasised that job opportunities were key to improving their lives and future prospects. However, there was a shared perception that securing stable, fixed-income employment remained a distant and often unattainable goal:

‘What I’m saying is, there is a need for a public policy for young adults to help them to get a job. We try everything. We need to earn money’ (J, male, beneficiary, 19 years old, Sapopemba).

### Theme 4: limitations of BFP and opportunities for improvement

#### ‘What I perceive in this neighbourhood is the policy of misery’

Focus groups participants considered the BFP important but did not associate it with increased opportunities and quality of life for young adults, or with improvements in their education and employability. The BFP was perceived just as one of the survival strategies that helped to access other resources, which could be related to formal or informal work:

‘When we think about young adults, they need to have fun, take professional courses, buy clothes, cell phones … It may be that Bolsa Família helps, but the family focuses on the issue of food’ (L, female, provider, 35 years old, Vila Maria).

Young adults confirmed that their families primarily use BFP funds to purchase food. However, in some cases, the money also supported purchases that boosted the young adults’ self-esteem. For instance, some mothers reported using the funds to help their children feel more confident at school by providing clothing, shoes or a mobile phone. Given the limited amount of the benefit, mothers with multiple children often alternated which child would receive these items:

‘Sometimes she [my mother] buy different things, those things that money couldn’t afford, then … Crackers … something tastier than just rice and beans; when there was a little leftover, clothes too’ (S, female, beneficiary, 18 years old, Sapopemba).

However, the context of high poverty affects young adults deeply. The fact that the BFP is not aimed directly at them, but seeks primarily to fight hunger, may prevent changes in future life chances of young adults. As mentioned by some young adults, the absence of public policies, the lack of resources to develop life projects, the social context of poverty and violence and the lack of opportunities in the neighbourhoods result in a complex situation in which the BFP alone represents a low possibility of generating substantial changes in their lives:

‘What I see in this neighbourhood is the politics of misery. This poverty policy is here in the neighbourhood, in all needs: education, health, assistance, in everything. No matter how much sacrifice we make, the policy of misery is established here. This affects the life of the woman who is there, that will suffer a lot of violence, because the lack of things inside the house brings disharmony, the drug addiction in the house brings disharmony, so this youth will be filled with this, with this misery, it’s just the “no” he’s had all his life to get somewhere. He will stand out in the drug trafficking, which represents immediate money, quick money, but which later becomes a snowball in his life. And then we don’t have this youth because he dies, or he dies from an overdose, or he dies by the police, or he dies by drug trafficking, somehow, he will die. So, we don’t see this young man having dreams’ (E, female, provider, 43 years old, Sapopemba).

Finally, although conditionalities were included in the topic guide because these were initially considered an important aspect of the BFP, young adults demonstrated limited knowledge about them and providers did not highlight these issues. As a result, conditionalities did not emerge as a relevant topic in their discourse.

## Discussion

Our study highlights the complexity of poverty in shaping the subjectivity of young adults in vulnerable situations across various outskirts of São Paulo city. Interestingly, poverty emerges both as an aspect of identity – ‘we are humble’ – and as a condition that ‘kills us from within’. This duality manifests in several ways: (a) the loss of autonomy and inability to envision life goals beyond mere survival; (b) anxiety surrounding the pursuit of the first job, which is seen as the primary means of improving life opportunities; (c) disillusionment with government actions; (d) a sense of powerlessness in the face of social inequality; and (e) suffering caused by violence and hunger. This vulnerable social context fosters a perception of limited opportunity and deep hopelessness.^38,39^ Notably, the BFP was not perceived as a policy that, in isolation, would directly impact the mental health of young adults. This finding aligns with recent quantitative studies conducted in Brazil^31^ and other LMICs.^20,24^

Several hypotheses can be proposed to explain these findings. First, the BFP primarily targets mothers rather than directly addressing the needs of young adults. Second, the funding provided by the programme is often considered too low and is primarily used to purchase food and meet other immediate needs. Additionally, previous research has emphasised the need to incorporate a specific mental health component within cash transfer programmes to achieve sustainable, long-term impacts.^40^ In this context, it remains challenging to evaluate the effects of income transfer policies alone in terms of changing behaviours or promoting significant improvements in quality of life.^41^ In addition to that, it’s important to remember that the number of studies evaluating the impact of the BFP on the mental health of Brazilian users is quite limited. Two of these show positive results, such as a decreased suicide risk among adults^21^ and an improvement in psychosocial health among children aged 7–17 years.^30^

Our results show that job opportunities were the central component of the young adults’ expectations. They expressed anguish, translated into a feeling of ‘outrage’, which is a consequence of the reproduction of poverty and ‘lack of opportunities’. Young adults discussed mental health in relation to concrete situations, such as job interviews and conversations with public authorities. Service providers and young people used the term ‘anxiety’ to describe insecurities about professional lives, hopelessness and frustration. Young adults had difficulties finding a different, broader perspective on future job and income prospects than their families and community; these are, in most cases, precarious. The word ‘hopelessness’ was frequently used by the young adults. The phenomenon of hopelessness among young adults resident in economically disadvantaged neighbourhoods is a pressing issue, albeit not a new one. In the face of uncertain prospects, some individuals lose hope, potentially leading to consequences such as fatalism and pessimism.^42^

Young adults emphasised that the low value of the cash transfer could not achieve significant changes in purchasing power, access to services and facilities and, as consequence, improvement in their life chances. This is in line with previous findings indicating that the amount allocated to families in CTPs needs to be substantial to bring broader impacts, including positive effects on the mental health of beneficiaries.^23,43,44^

Our data collection with providers indicated that violence was experienced in daily life in its multiple dimensions, which is consistent with the vast literature on violence in Brazilian cities and its impact on the lives of inhabitants, particularly young adults.^11^ Experience of violence is associated with poor mental health, including feelings of depression, sadness and helplessness.

Study participants highlighted a perceived gap in the expansion and coordination of public policies addressing mental health, education and employment, which hampers the evaluation of their impact on the life prospects of future generations. This concern aligns with evidence from previous studies, which emphasise that integrated interventions targeting adolescent outcomes yield greater benefits when implemented together rather than in isolation. Such integrated approaches include initiatives such as creating safe living environments, ensuring timely access to healthcare and strengthening protective factors essential to adolescent well-being.^45^

Social protection measures, such as BFP, play an important role by providing a minimum level of food security to poor families during crises.^29^ The scourges of poverty and poor mental health of young adults need to be addressed concurrently,^40,46^ if the life chances of young adults are to move further away from the cycle of poverty, poor mental health and socioeconomic disadvantage.^27^

This study adds new knowledge about the role of the BFP in the lives of vulnerable young people, but some limitations must be pointed out. All participants were from São Paulo city, which limits the generalisation of results to other regions in Brazil. For example, São Paulo is a city with the highest cost of living in the country. In addition, despite the focus of the study being on mental health, the analysis of these aspects was limited because the young adults emphasised themes such as work and education, indicating the complexity of the mental health theme in the studied context. In this sense, it was not possible to compare profiles that presented a higher risk for depression and anxiety with participants considered healthy during the study.

Despite these limitations, the study enhances understanding of how the BFP is perceived by young adults and professionals working with them. The findings underscore that, while the BFP plays an essential role in meeting basic needs, it is insufficient on its own to improve long-term life prospects. Integrating complementary policies – particularly those focused on employment, income generation and mental health – could foster greater autonomy and improve future opportunities for young people living in poverty in Brazil.

## Data Availability

The data that support the findings of this study are available from the corresponding author, C.S.P., on reasonable request.
